# Entecavir versus tenofovir for prevention of hepatitis B virus-associated hepatocellular carcinoma after curative resection: study protocol for a randomized, open-label trial

**DOI:** 10.1186/s13063-023-07742-x

**Published:** 2024-01-05

**Authors:** Li-Xin Pan, Yi-Yang Wang, Zhong-Hai Li, Jia-Xi Luo, Kun-Jun Wu, Zhen-Xiu Liu, Pei-Sheng Wu, Kang Chen, Liang Ma, Xiao-Hui Fan, Jian-Hong Zhong

**Affiliations:** 1 Hepatobiliary Surgery Department, Guangxi Medical University Cancer Hospital, Nanning, China; 2 Hepatobiliary Surgery Department, the First People’s Hospital of Qinzhou, Qinzhou, China; 3 Hepatobiliary Surgery Department, The Second Affiliated Hospital of Guangxi Medical University, Nanning, China; 4 Department of Microbiology, School of Preclinical Medicine, Guangxi Medical University, Nanning, China; 5 Key Laboratory of Early Prevention and Treatment for Regional High Frequency Tumors (Guangxi Medical University), Ministry of Education, Nanning, China; 6 Guangxi Key Laboratory of Early Prevention and Treatment for Regional High Frequency Tumors, Nanning, China

**Keywords:** Hepatocellular carcinoma, Hepatitis B virus, Entecavir, Tenofovir disoproxil fumarate

## Abstract

**Background:**

Entecavir and tenofovir disoproxil fumarate (TDF) are standard first-line treatments to prevent viral reactivation and hepatocellular carcinoma (HCC) in individuals chronically infected with the hepatitis B virus (HBV), but the long-term efficacy of the two drugs remains controversial. Also unclear is whether the drugs are effective at preventing viral reactivation or HCC recurrence after hepatectomy to treat HBV-associated HCC. This trial will compare recurrence-free survival, overall survival, viral indicators and adverse events in the long term between patients with HBV-associated HCC who receive entecavir or TDF after curative resection.

**Methods:**

This study is a randomized, open-label trial. A total of 240 participants will be randomized 1:1 into groups receiving TDF or entecavir monotherapy. The two groups will be compared in terms of recurrence-free and overall survival at 1, 3, and 5 years after surgery; adverse events; virological response; rate of alanine transaminase normalization; and seroreactivity at 24 and 48 weeks after surgery.

**Discussion:**

This study will compare long-term survival between patients with HBV-associated HCC who receive TDF or entecavir monotherapy. Numerous outcomes related to prognosis will be analyzed and compared in this study.

**Trial registration:**

ClinicalTrials.gov NCT02650271. Registered on January 7, 2016.

## Introduction

### Background and rationale

Chronic infection with the hepatitis B virus (HBV), which affects more than 290 million people worldwide [1], is one of the strongest risk factors for cirrhosis and hepatocellular carcinoma (HCC). Entecavir and tenofovir disoproxil fumarate (TDF) are widely recommended as standard first-line treatments capable of slowing progression of cirrhosis and reducing the risk of liver failure and onset of HCC [2, 3]. Some studies have shown that monotherapy with entecavir or TDF can significantly reduce viral load in plasma and improve liver function without eliciting drug resistance or serious adverse events [4–6]. In most parts of the world, generic formulations of both drugs are widely available at relatively low prices.

While strong evidence exists that either monotherapy can prevent viral reactivation and HCC in the short term, whether it does so in the long term is less clear. Stopping the medication leads in many cases to reactivation of HBV infection and increased risk of HCC, even in patients whose viral load or hepatitis B surface antigen has been undetectable for years [2, 3, 7]. We are unaware of any randomized studies examining the ability of entecavir or TDF to prevent cirrhosis or HCC in the long term in patients chronically infected with HBV, and the numerous prospective or retrospective cohort studies in the literature have come to divergent conclusions. For example, for patients receiving initial treatment, some studies found TDF to be significantly better than entecavir at preventing HCC [8–10], but other studies involving first- or second-line treatment found them to be similarly effective for this outcome [11–14]. Even meta-analyses have come to divergent conclusions: several indicated the superiority of TDF for preventing HCC [15–17], while another reported similar efficacy for the two drugs [18]. These considerations highlight the need for a large, rigorously designed trial to compare the two monotherapies with long follow-up.

Similarly, the literature is unclear about how well either monotherapy prevents viral reactivation or recurrence of HCC after patients with HBV-associated HCC have undergone curative hepatectomy. The drugs are often given prophylactically to such patients after hepatic resection in order to inhibit viral replication and thereby reduce risk of HCC recurrence. Indeed, we and others have shown that preoperative levels of HBV DNA or surface antigen HBsAg are associated with risk of HCC recurrence [19, 20], and that entecavir monotherapy can reduce this risk [21, 22]. Some retrospective studies [23, 24] and a meta-analysis [25] have shown TDF to protect against HCC recurrence better than entecavir, while other studies have concluded the two drugs to be similarly effective for this outcome [26, 27]. The above retrospective studies did not find differences between entecavir and TDF in virological response, serological reaction, or adverse reactions.

### Objectives

To clarify and compare the long-term efficacies of the two monotherapies, we have launched a randomized trial to compare monotherapy with entecavir or TDF for preventing viral reactivation and HCC recurrence in the short and long term after hepatic resection to treat HBV-associated HCC.

### Trial design

This study is a phase 3 randomized, open-label, superiority trial. Participants will be randomly divided into two groups in a ratio of 1:1, one of which will receive TDF monotherapy and the other will receive entecavir monotherapy during the peri- and postoperative period. The primary outcome of the study is the rate of recurrence-free survival at 1, 3 and 5 years after curative hepatectomy. The secondary outcomes are the rate of overall survival at 1, 3, and 5 years; adverse reactions; virological response; rate of alanine transaminase normalization; and seroreactivity at 24 and 48 weeks after surgery.

## Methods

### Study setting

This study will be conducted at the Guangxi Medical University Cancer Hospital, the First People’s Hospital of Qinzhou, and the Second Affiliated Hospital of Guangxi Medical University. These three hospitals are all located in Guangxi province, a high incidence area of HCC in China.

### Eligibility criteria

To be enrolled in the study, participants must be 18–75 years old, they must have tested positive for HBsAg and/or HBV DNA at admission, they must have undergone curative hepatectomy at one of the study sites, and their diagnosis of HCC must have been confirmed by postoperative histopathology. Curative resection is defined based on intra- and postoperative criteria [28]. Participants must also have scored 0 or 1 on the ECOG Performance Status Scale at admission. They must be aware of their condition and able to provide written consent.

Potential participants will not be enrolled if they tested positive at admission for antibodies against hepatitis C virus or human immunodeficiency virus, or if they were receiving any anti-HBV therapy before enrollment, such as interferon, entecavir, TDF, adefovir dioxide, or lamivudine. They will also be excluded if they have a history of other malignancies or serious diseases within the previous five years, a history of organ transplantation or drug allergy, autoimmune disease or history of long-term use of glucocorticoids. Pregnant or lactating women will not be considered for enrollment. Participants who fail to comply with treatment or complete follow-up will be excluded from the study.

### Recruitment

Eligible inpatients of the Guangxi Medical University Cancer Hospital, the First People’s Hospital of Qinzhou, and the Second Affiliated Hospital of Guangxi Medical University will be included into this study. The benefits and safety of the treatment plan in this study for patients will be explained to secure continuous recruitment.

### Who will take informed consent?

Informed consent will be completed by the participants during the perioperative period after they get understand to the benefits and safety of this study, and it will be delivered to the trained research staff in person.

### Additional consent provisions for collection and use of participant data and biological specimens

There are no additional consent provisions since the participant data will only be used for the purpose of the current trial, but not for future use in ancillary studies. Blood will be collected to assess liver function, renal function, HBV DNA levels, and other outcomes in the trial.

### Interventions

#### Explanation of the choice of comparators

Entecavir and TDF are standard first-line treatments for individuals chronically infected with HBV in clinical practice, and patients can obtain both drugs at relatively low prices. Although the short-term efficacies of either monotherapy with entecavir or TDF in preventing viral reactivation and HCC have been certified, the long-term efficacies in preventing tumor recurrence for patients with HBV-associated HCC after curative hepatectomy are still controversial. Therefore, this study aims to compare the long-term efficacies of the two monotherapies.

#### Description of interventions

Once the first blood test is positive for HBsAg or HBV DNA, participants will be prescribed 300 mg/day of oral TDF or 0.5 mg/day of oral entecavir for long-term administration. After curative hepatectomy, patients will return to the hospital for follow-up examinations according to standard practices at the study sites.

#### Criteria for discontinuing or modifying allocated interventions

Titer of HBV DNA in blood will be determined every 12 weeks in all participants. If the titer of HBV DNA has not decreased significantly by 3 months after surgery, which suggests drug resistance, patients who have been receiving 300 mg/day of oral TDF will also be given 0.5 mg/day of oral entecavir, while patients who have been receiving 0.5 mg/day of oral entecavir will switch to only 300 mg/day of oral TDF. Otherwise, patients will continue on the same medication as at enrollment. All participants will receive antiviral therapy until death or the end of the study, given that there are no consensus guidelines on the length of antiviral treatment for patients with HBV-associated HCC. If serum HBV DNA has not decreased after one month of conversion therapy, patients will be switched to tenofovir alafenamide fumarate.

#### Strategies to improve adherence to interventions

The intervention plans in this study belong to the clinical routine treatment, and both the incidence of serious adverse reactions and treatment costs are low. This study will adhere to the relevant regulations of medical ethics and Good Clinical Practice (GCP). Investigators will fully communicate with patients to improve compliance and minimize shedding and missing follow-up cases as much as possible.

#### Relevant concomitant care permitted or prohibited during the trial

Repeat hepatic resection, ablation, systemic chemotherapy, radiation therapy, targeted drugs, immunotherapy, and other anti-tumor treatments for recurrent HCC are permitted during the study [29–31]. At the investigators’ discretion, participants will also be allowed to continue long-term medications for chronic diseases such as hypertension or diabetes.

#### Provisions for post-trial care

The study will not provide specific provisions for post-trial care. Recommendations and referrals will be given by licensed clinical study staff to the participants who asked for additional clinical care.

### Outcomes

Patients will receive routine follow-up every month for the first three months, every two months for six months to two years, then every six months thereafter. At each follow-up, tests will be performed to assess liver function, HBV DNA load, virological response, serum alpha fetoprotein level, and other hematological indicators. Each follow-up will also involve liver ultrasonography as well as contrast-enhanced computer tomography of the chest and abdomen or magnetic resonance imaging of the abdomen (Table 1).

**Table 1:** Study assessment measures

Assessment measures	Preoperation	Postoperative day 5	One month after surgery	Two months after surgery	Three months after surgery	Six months after surgery	Every six months until HCC recurrence /metastasis or death
Blood routine examination	x	x	x	x	x	x	x
Urine routines	x	x	x	x	x	x	x
Stool routine examination	x	x	x	x	x	x	x
Liver function	x	x	x	x	x	x	x
Renal function	x	x	x	x	x	x	x
Myocardial enzyme	x	x	x	x	x	x	x
Hepatitis B virological test^1^	x		x	x	x	x	x
Coagulation function	x	x	x	x	x	x	x
Lipid level^2^	x		x	x	x	x	x
Alpha fetoprotein	x		x	x	x	x	x
Des-gamma-carboxy prothrombin	x		x	x	x	x	x
Hepatits C antibody	x						
Human immunodeficiency virus antibody	x						
• Blood HCG test^3^	x						
Electrocardiogram	x		x	x	x	x	x
Abdominal ultrasonography	x		x	x	x	x	x
Chest and upper abdomen enhanced MRI/CT	x		x	x	x	x	x

*CT* Computed tomography, *HCC* Hepatocellular carcinoma, *MRI* Magnetic resonance imaging, *RFS* Recurrence-free survival, *OS* Overall survival

1. Including hepatits B surface antigen, hepatits B surface antibody, hepatits B e antigen, hepatits B e antibody, hepatits B c antibody, and hepatits B virus DNA

2. Including triglyceride, cholesterol, high-density lipoprotein, and low-density lipoprotein

3. Women of childbearing age

The primary outcome is recurrence-free survival, defined as the time between the randomization date and first occurrence of HCC recurrence, as detected by imaging. Recurrence can be extrahepatic or new intrahepatic. The secondary outcomes of this trial include overall survival, defined as the time between the randomization date and death. If the participant is alive at the end of the study or is lost to follow-up, the last follow-up visit will be used. Other secondary outcomes are virological response, defined as titer of HBV DNA; rate of alanine transaminase normalization; seroreactivity at 24 and 48 weeks after resection, defined as the presence of HBsAg or HBV e antigen; frequency of treatment-related adverse events, as assessed using the Common Terminology Criteria for Adverse Events (version 5.0) [32]; and treatment tolerability, measured in terms of the proportion of patients completing the planned treatment.

### Participant timeline

**Fig. 1:**
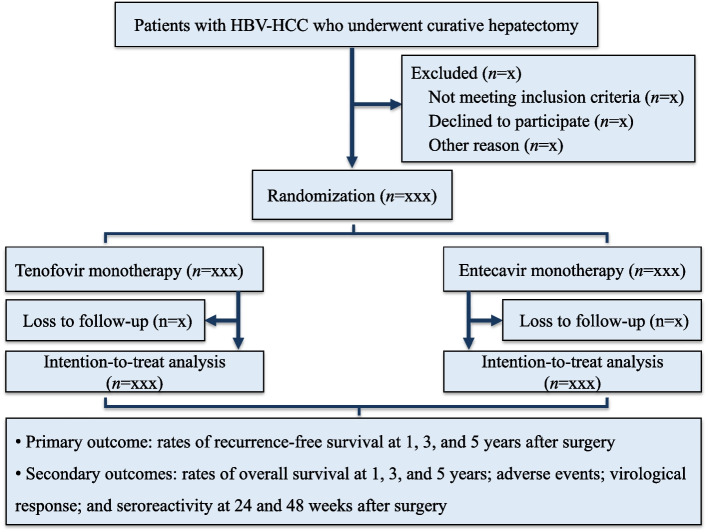
Participant flowchart. HBV, hepatitis B virus; HCC, hepatocellular carcinoma

The participant timeline of this study is shown in Fig. 1.

### Sample size

We estimated using PASS 15.0.5 that we would need at least 108 subjects in each group, assuming 3-year rates of recurrence-free survival of 64% among those on entecavir monotherapy [23] and anticipated 79% among those on TDF monotherapy, enrollment lasting two years and follow-up lasting five years. This sample should be able to detect a significant difference between the groups with 90% power and bilateral α = 0.05. We increased the total number from 216 to 240 to compensate for 10% loss to follow-up.

### Assignment of interventions: allocation

#### Sequence generation

Statisticians will generate randomization cards using stratified randomization according to sex, tumor stage, and preoperative titer of HBV DNA.

#### Concealment mechanism

Allocation information will be kept in sealed envelopes. The envelopes and internal original records will be kept by the department for future reference.

#### Implementation

After participant eligibility has been confirmed, a specially designated person will open the sealed envelope to obtain the random number, which will serve to assign patients 1:1 to either group.

### Assignment of interventions: blinding

#### Who will be blinded

Study investigators and participants, but not data analysts, will be aware of group allocation.

#### Procedure for unblinding if needed

Under special circumstances, the data on informed consent forms, allocation information, and paper-based case report forms (CRFs) may be shared with appropriate personnel in the event of a medical emergency or audit by regulatory agencies.

### Data collection and management

#### Plans for assessment and collection of outcomes

Assessment measures and corresponding timing are listed in Table 1. Research staff conducting the pre- and post-treatment, and follow-up assessments will receive specific training. Study instruments used for the assessments will be maintained daily according to the requirements of the corresponding department.

#### Plans to promote participant retention and complete follow-up

This study will not generate extra examination and treatment costs for the participants. Before participants are enrolled, investigators will explain the procedures, benefits, and risks of the trial as well as the participants’ rights, including the right to withdraw from the trial at any time for any reason. Individuals will be enrolled in the study only after signing written informed consent.

#### Data management

Study investigators will complete CRFs for all participants. The CRFs will be reviewed by a clinical research assistant, after which the investigators will sign the forms and the data can no longer be modified. Two investigators will separately enter all participant data into a central database to ensure data accuracy. Once the data have been checked, they will be locked as unmodifiable in the database. Data analysts will then perform all statistical analyses, and the results will be given to investigators, who will write the complete trial report.

The central database will be protected from unauthorized access. Completed CRFs, signed informed consent forms, randomization envelopes, as well as the study protocol and eventual amendments will be archived in a secure location at the study sites for at least five years after completion of the trial.

#### Confidentiality

Informed consent forms, allocation numbers, and CRFs will be stored in a secure location to which only authorized personal have access.

### Plans for collection, laboratory evaluation, and storage of biological specimens for genetic or molecular analysis in this trial/future use

This study will not collect biological specimens for genetic or molecular analyses.

### Statistical methods

#### Statistical methods for primary and secondary outcomes

All statistical analyses will be conducted in a blinded manner. Data will be reported as mean ± SD, and inter-group differences in continuous variables will be assessed for significance using Student's *t* test, while differences in categorical variables will be assessed using the chi-squared test. Survival curves will be calculated using the Kaplan–Meier method and compared using the log-rank test. Cox regression will be applied to identify factors associated with primary and secondary outcomes.

#### Interim analyses

An interim analysis will be performed after 50% of subjects have completed the 3 months study period. The timing of additional interim analyses will be determined by the Data and Safety Monitoring Board. The committee will not have executive power to stop the trial or modify treatment but can make a recommendation for the former or latter. The Data and Safety Monitoring Board will provide reports of its observations and recommendations to the Trial Management Group and lead Ethics Committee.

#### Methods for additional analyses (e.g., subgroup analyses)

Subgroup analysis will also be conducted according to sex, tumor stage, cirrhosis, preoperative titer of HBV DNA and other factors judged relevant to prognosis.

#### Methods in analysis to handle protocol non-adherence and any statistical methods to handle missing data

Data analysis of this study will according to the intention-to-treat principle. Efforts are made to follow-up participants who dropped out from treatment in order to obtain more evaluation indexes and recorded in the CRFs. The last assessment data will be applied for patients who lost to follow-up.

#### Plans to give access to the full protocol, participant-level data, and statistical code

Full protocol, participant data, and statistical code of this study will be accessed for grant public after the full paper of this study is published.

### Oversight and monitoring

#### Composition of the coordinating center and trial steering committee

The trial management will be conducted under the auspices of the Guangxi Medical University Cancer Hospital in accordance with the current standards of GCP in China and the protection of subjects’ rights and interests in accordance with the Declaration of Helsinki.

#### Composition of the data monitoring committee, its role, and reporting structure

The data monitoring committee is composed of the GCP of Guangxi Medical University Cancer Hospital. The office staff of GCP will be in regular contact with the researchers to follow up on the progress of the trial and address any questions they may have. The CRF is reviewed by the staff to check for consistency with the study protocol, data consistency, missing data, and scheduling. If the patient consents, the investigator will need to send a copy of the signed informed consent to the office of GCP for review. When there is a problem with the data, the office of GCP will ask the investigator to provide a data description sheet to supplement or explain the data inconsistency, missing data, etc. Any significant issues identified during monitoring are reported, as appropriate, to the trial management team and the relevant authorities, including serious breaches of the GCP and/or the study protocol. The investigator shall be subject to monitoring, auditing, ethical review and management inspections related to the trial, and shall provide the original data and documentation of the trial.

#### Adverse event reporting and harms

Adverse events will be assessed using the Common Terminology Criteria for Adverse Events (version 5.0) [32]. Adverse events and serious adverse events (SAE) will be recorded including name of adverse event, start time, end time, degree, correlation with study drug, and accompanying treatments. In addition to complying with the requirements of the SAE report of the department, it is also necessary to report to the ethics committee of the principal investigator within 24 h of being informed of the occurrence of SAE. Follow up will carry on until the event is resolved or the condition of the participant return to baseline.

#### Frequency and plans for auditing trial conduct

Trial Management Group will auditing trial conduct every two years.

#### Plans for communicating important protocol amendments to relevant parties (e.g., trial participants, ethical committees)

Principal investigator will conduct amendments to the study protocol and then send to the ethics committee and co-investigators. Update of the trial registry will be performed simultaneously.

#### Dissemination plans

This study has been registered at www.clinicaltrials.gov (NCT02650271), and the original, anonymized data will be published at www.researchdata.org.cn. Data analysis from this study will be published at relevant conferences and in peer-reviewered publications. Requests for unpublished data can be submitted to the corresponding authors and will be honored insofar as relevant ethics and confidentiality rules allow.

## Discussion

Entecavir and TDF are both standard first-line treatments for patients with chronic hepatitis B. However, the long-term efficacy of monotherapy with entecavir or TDF in preventing tumor recurrence for patients with HBV-related HCC after curative hepatectomy are still controversial and need to be further verified.

This study will analyze numerous outcomes related to prognosis, including adverse events, virological response, rate of alanine transaminase normalization, and seroreactivity. The results of this study will help to advance our understanding of the long-term efficacy of entecavir or TDF monotherapy. A randomized controlled trial with small sample size comparing the efficacy of TDF (*n* = 74) and entecavir (*n* = 74) monotherapy for patients with HBV-related HCC after curative treatment found TDF therapy was associated with a significantly lower risk of tumor recurrence than entecavir therapy [33]. Our randomized trial with large sample size will provide more stronger evidence about the efficacy of entecavir or TDF monotherapy for patients with HBV-related HCC after curative resection. In general, the larger the sample size and the longer the follow-up, the more reliable the results. We had planned to revise the reference [27] for the sample size calculation, expecting to enroll 638 cases, but did not get approval from the Ethics Committee of Guangxi Medical University Cancer Hospital. The Ethics Committee is of the view that since this study is not directly supported by sufficient funding, there is a risk that if the sample size is further expanded, it may not be successfully completed in accordance with the intended objectives.

A limitation worth noting is that participants will know their assigned treatment may lead some to request conversion of their treatment, triggering their exit from the study.

### Trial status

The sample size was anticipated as 240 involving in one center in the protocol version 1.0. In this protocol version 1.1, more other liver centers would be included. This study refers to protocol version 1.1 (June 15, 2023). Recruitment began in July 2021. The trial is ongoing and recruiting participants. Recruitment is anticipated to be completed on 31 December 2023.

## Data Availability

The principal investigator, Jian-Hong Zhong, MD, PhD, will have access to the final trial dataset. Any data required to support the protocol can be supplied on request.

## Abbreviations

CRF: Case report form
HBV: Hepatitis B virus
HCC: Hepatocellular carcinoma
GCP: Good clinical practice
SAE: Serious adverse events
TDF: Tenofovir disoproxil fumarate
